# Maternal and neonatal characteristics associated with clinical outcomes of TOLAC from 2012–20 in the USA: Evidence from a retrospective cohort study

**DOI:** 10.1016/j.eclinm.2022.101681

**Published:** 2022-09-28

**Authors:** Hanxu Shi, Siwen Li, Jin Lv, Harry H.X. Wang, Qingxiang Hou, Yinzi Jin

**Affiliations:** a Department of Global Health, School of Public Health, Peking University, Beijing 100191, China; b Institute for Global Health and Development, Peking University, Beijing 100191, China; c School of Public health, University of Hong Kong, Hong Kong; d Central Laboratory of Research Department, the PLA Rocket Force Characteristic Medical Centre, Beijing 100088, China; e School of Public Health, Sun Yat-Sen University, Guangzhou 510080, China; f Usher Institute, Deanery of Molecular, Genetic and Population Health Sciences, The University of Edinburgh, Edinburgh EH8 9AG, UK; g Gynaecology and Obstetric Department, the PLA Rocket Force Characteristic Medical Centre, Beijing 100088, China

**Keywords:** Trial of labour after caesarean (TOLAC), Maternal and neonatal characteristics, Maternal and neonatal morbidity, TOLAC, trial of labour after caesarean, aOR, adjusted odds ratio, aPR, adjusted prevalence ratio, NCHS, National Centre for Health Statistics, BMI, body mass index, ICU, intensive care unit, NICU, neonatal intensive care unit

## Abstract

**Background:**

The risks of a few maternal and/or neonatal morbidities are higher with the trial of labour after caesarean (TOLAC) owing to unplanned caesarean delivery. Thus, it is imperative to consider the trade-off between the risk of side effects and the potential benefits before TOLAC utilisation and whether TOLAC should be provided to women with specific characteristics related to previous caesarean delivery. We aimed to investigate maternal and neonatal characteristics associated with TOLAC utilisation, compare maternal and/or neonatal morbidities in TOLAC women with women who chose planned caesarean deliveries, and assess specific characteristics related to maternal and/or neonatal morbidities in women with TOLAC utilisation.

**Methods:**

In this retrospective cohort study, we used nationwide, linked birth and infant death data in the United States between 2012 and 2020, which covers all 50 states in the US. Poisson regression models using generalised estimating equations yielded adjusted prevalence ratios (aPRs) with 95% confidence intervals (CIs) of TOLAC utilisation and unsuccessful TOLAC by maternal and neonatal characteristics. Logistic regression models using generalised estimating equations yielded adjusted odds ratios (aORs) with 95% CIs of maternal and neonatal morbidities. Statistical analysis was performed from February 2022 to July 2022.

**Findings:**

The sample included 4,898,441 women with mean (SD) maternal age years (5.4 years; range 13–50). Several specific maternal and neonatal characteristics were significantly associated with unsuccessful TOLAC, although women with TOLAC utilisation were associated with significantly lower risks of maternal unplanned hysterectomy (aOR, 0.60; 95% CI, 0.60–0.61), admission to intensive care (aOR, 0.84; 95% CI, 0.84–0.85), and neonatal seizures (aOR, 0.80; 95% CI, 0.74–0.84). In women who attempted TOLAC, advanced maternal age, higher maternal body mass index, more than 2 previous caesarean deliveries, having maternal co-morbidities and fetal malpresentation increased the likelihood of maternal and neonatal morbidities.

**Interpretation:**

When utilising TOLAC, specific maternal and neonatal characteristics in pregnant women should be considered in conjunction with the potential benefits of TOLAC in preventing maternal and neonatal morbidities.

**Funding:**

This study is funded by the Clinical Medicine Plus X - Young Scholars Project, Peking University, the Fundamental Research Funds for the Central Universities (No: PKU2022LCXQ008).

**Research in context**

**Evidence before this study**

We searched PubMed, Web of Science, and the Cochrane Library using the keywords “trial of labour after caesarean”, “TOLAC”, “maternal morbidity”, “neonatal morbidity”, “maternal characteristics”, and “neonatal characteristics”, with the recent 10 years’ restriction. Up to June 30, 2022, we yielded 3 relevant studies. Previous studies only focused on predictors associated with TOLAC utilisation, rather than investigating specific maternal/neonatal characteristics associated with maternal and neonatal morbidities among women who utilised TOLAC.

**Added value of this study**

The present study used the nationwide birth cohort datasets in the United States between 2012 and 2020, which included 4,898,441 women covering all 50 states.

The present study revealed several maternal and neonatal characteristics associated with unsuccessful TOLAC, although TOLAC utilisation was beneficial to lowering the risk of maternal unplanned hysterectomy, admission to ICU, and surfactant when compared with the planned caesarean delivery. In women who attempted TOLAC, advanced maternal age, higher maternal BMI, more than 2 previous caesarean deliveries, having maternal co-morbidities and birth malpresentation increased the likelihood of maternal and neonatal morbidities.

**Implications of all the available evidence**

Our findings provide evidence-based guidance to identify women who are suitable for attempting TOLAC, so as to prevent maternal and neonatal morbidities for TOLAC women with specific maternal/neonatal characteristics.

Alt-text: Unlabelled box

## Introduction

Alarmingly, the annual incidence of caesarean delivery in the United States (US) has increased dramatically, from 23.5% in 1988 and reaching 31.8% in 2020.^1^^,^^2^ Repeated caesarean delivery causes concerns among health care providers and patients as this is associated with an increased likelihood of maternal and/or neonatal morbidity.^3^ The World Health Organization (WHO) and National Institutes of Health have held consensus conferences to consider trial of labour after previous caesarean delivery (TOLAC) as a reasonable strategy for reducing the rate of caesarean delivery.^4^^,^^5^ Previous studies have confirmed the success of TOLAC in minimising adverse maternal and/or neonatal morbidity including surgical infections, pelvic adhesions, and morbidly adherent placenta.^6^^,^^7^ Despite the recent Practice Bulletin by the American College of Obstetricians and Gynecologists recommending that most women with one previous caesarean delivery are candidates for and should be counseled about TOLAC utilisation,^8^ it is important to evaluate the likelihood of suffering from maternal and neonatal morbidities in women with TOLAC utilisation.

Attempting TOLAC is beneficial to quicker recovery, delivery without abdominal surgery, infection and blood loss reduction.^9^^,^^10^ Nevertheless, the association of TOLAC utilisation with maternal and/or neonatal morbidity has rarely been investigated in comparison with planned caesarean deliveries. Thus, health care providers (i.e., physicians, obstetricians) must accurately identify women who are more suitable to have TOLAC attempted, and in whom planned caesarean deliveries. Given the trade-off between the risk of maternal and/or neonatal morbidities from TOLAC utilisation and the planned caesarean deliveries, it is essential to compare these two groups in association with maternal and/or neonatal morbidities.

Previous studies have identified several factors related to TOLAC utilisation, including malpresentation in previous delivery, gestational age at birth, presence of medical conditions, and birth weight.^11^, ^12^, ^13^, ^14^ However, existing reports have rarely addressed the decision to choose TOLAC among women with history of caesarean deliveries regarding maternal and/or neonatal morbidities,^15^ especially data regarding the maternal and/or neonatal outcomes in women with TOLAC utilisation had more than 2 previous caesarean deliveries.^16^ Therefore, investigating the specific maternal and neonatal characteristics associated with TOLAC utilisation resulted in maternal and/or neonatal morbidities remains unknown.

To fill in these gaps, we aimed to use a retrospective birth cohort in the US to: 1) investigate maternal and neonatal characteristics associated with TOLAC utilisation and unsuccessful TOLAC in women with a history of at least one prior caesarean delivery; 2) assess the prevalence of maternal and/or neonatal morbidity in women with TOLAC utilisation and those with planned caesarean deliveries and its association between these two groups; 3) examine specific maternal and neonatal characteristics (i.e., maternal age, maternal race, gestational age, maternal body mass index, prenatal care, pregnancy intervals, number of prior caesarean deliveries, maternal previous disease, birth weight, fetal presentation) associated with maternal and/or neonatal morbidities among women who utilised TOLAC.

## Methods

### Study participants

The birth cohort used in this study linked birth and infant death datasets between 2012 and 2020 from the National Centre for Health Statistics (NCHS), which covers all 50 states in the US.^17^ This database is deidentified and publicly available and the NCHS assumes responsibility for ethical data collection and publication. More details regarding the database are available from the NCHS.^17^ We limited sample to women at least had one prior caesarean delivery, had live singleton births, no congenital anomalies, and had successful linkage across maternal medical records, neonatal medical records, and birth certificate. A woman was identified as using TOLAC if her delivery method was noted as vaginal delivery or trial of labour attempted, or if the International Classification of Disease Tenth Revision (ICD-9) procedure code on the maternal hospital discharge record indicated a process of labour (Table S1).^18^ Women who did not meet these criteria for TOLAC and finally delivered births through repeated caesarean sections were assumed to have undergone a planned caesarean delivery.^18^ Ethical approval of this study was waived.

### Outcome measures

Outcome measures included maternal and/or neonatal morbidities. Maternal morbidities were identified from the medical records as an individual indicator for several maternal morbidities. This individual indicator followed the definition of the US Centre for Disease Control and Prevention and encompassed 16 diagnoses and five procedures suggestive of severe maternal complications, including maternal transfusion, perineal laceration, uterine rupture, unplanned hysterectomy, and admission to intensive care.^19^^,^^20^ We also identified significant neonatal morbidities from the birth certificate, including the presence of any assisted ventilation, admission to the neonatal intensive care, surfactant, antibiotic therapy, seizures.

### Maternal and/or neonatal characteristics

To investigate maternal and neonatal characteristics that are associated with women with TOLAC utilisation and unsuccessful TOLAC, as well as assess maternal and neonatal morbidities among attempted TOLAC women and those chose planned caesarean deliveries from multiple data sources were used, including: 1) maternal age, maternal race, prenatal care beginning, number of previous caesarean delivery, pre-pregnancy diabetes, gestational diabetes, pre-pregnancy hypertensive disorders, gestational hypertensive disorders, preterm, eclampsia, gonorrhea, syphilis, and hepatitis B based on maternal medical records^21^; 2) gestational age, maternal weight gain, and delivery methods derived from labour and delivery records, admission history, or physical examination^21^; 3) body mass index, calculated as pre-pregnancy weight in kilograms divided by height in meters squared, and categorised into six groups according to the WHO classification: underweight (<18.5 kg/m^2^), normal weight (18.5–24.9 kg/m^2^), overweight (25.0–29.9 kg/m^2^), obesity (≥30 kg/m^2^); 4) fetal presentation, and birth weight which were obtained from the birth certificate.

### Statistical analysis

We calculated the proportions of TOLAC utilisation and unsuccessful TOALC by maternal and neonatal characteristics, proportions of maternal and neonatal morbidities by specific maternal and neonatal characteristics in women with TOLAC utilisation, and proportions of maternal and neonatal morbidities in TOLAC utilisation and planned caesarean delivery. We used Poisson regression models with robust standard errors calculation by using generalised estimation equations accounting for birth place clustering effects to determine maternal and neonatal characteristics that were associated with the likelihood of women with TOLAC utilisation and unsuccessful TOLAC.^22^ We generated logistic regression models with robust standard errors calculation by using generalised estimation equations accounting for birth place clustering effects to investigate the associations of specific maternal and neonatal characteristics with maternal and neonatal morbidities in women who attempted TOALC and to compare the differences of maternal and neonatal morbidities in women with TOLAC utilisation and those with planned caesarean deliveries.^23^ We also adjusted for the maternal and neonatal characteristics which were associated with maternal and neonatal morbidities, as they were common in the association of themselves, including maternal age, maternal race, smoking, maternal BMI, gestational weight gain, prenatal care beginning, pre-pregnancy/gestation diabetes, pre-pregnancy/gestation hypertensive disorders, preterm (more details in Figure S2). Missing rates were less than 1.5% for all other variables used in the present analyses (Table S2).

The sensitivity analysis was performed which we stratified samples into groups of TOLAC utilisation and planned caesarean delivery based on their estimated propensity score, and estimated an overall treatment effect by pooled the stratum-specific estimates.^24^ All two-sided P values less than 0.05 denoted statistical significance. All analyses were performed using R version 3.6.2 (The R Project for Statistical Computing, Vienna, Austria). Statistical analysis was performed from 11^th^ of February, 2022 to 2^nd^ of June, 2022.

### Role of the funding source

The study sponsor has no role in study design, data analysis and interpretation of data, the writing of manuscript, or the decision to submit the paper for publication. All authors had full access to all the data in the study and had final responsibility for the decision to submit for publication.

## Results

Between the Feb 11 and June 2, 2022, we included 4,898,441 women with a history of at least one prior caesarean delivery (Table 1), of which 867,843 (17.7%) utilise TOLAC. Among TOLAC women, 245,981 (28.3%) experienced an unsuccessful TOLAC (i.e., an unplanned caesarean delivery) (Figure S1).

**Table 1:** Sample characteristics in the whole sample during 2012–2020 in the US.

Characteristics	Overall^a^ (*N*=4,898,441)
**Maternal characteristics**	
**Maternal age, mean (SD), years**	30.7 (5.4)
**Maternal age, years, (%)**	
<25	14.0
25–34	60.5
≥35	25.5
**Maternal race, (%)**	
Non-Hispanic	73.8
Hispanic and Latino American	22.0
Other Hispanic	4.2
**Gestation age, weeks, (%)**	
<37	11.7
37–42	84.1
>42	4.2
**Maternal BMI, kg/m^2^, (%)**	
Underweight	2.0
Normal weight	33.9
Overweight	27.3
Obesity	36.8
**Gestation weight gain, pounds, (%)**	
<20	30.8
20–40	50.8
>40	18.4
**Maternal smoking, (%)**	
No	91.9
Yes	8.1
**Prenatal care beginning, month, (%)**	
0	4.0
1^st^–3^rd^	74.6
4^th^–6^th^	16.8
After 7^th^	4.6
**Number of previous caesarean, (%)**	
<2	92.2
≥2	7.8
**Maternal co-morbidities**	
Prepregnancy diabetes, (%)	1.6
Prepregnancy hypertensive disorders, (%)	2.8
Preterm, (%)	7.0
Eclampsia, (%)	0.3
Gonorrhea, (%)	0.3
Syphilis, (%)	0.1
Hepatitis B, (%)	0.3
**Neonatal characteristics**	
**Birth weight, grams, (%)**	
Underweight	6.6
Normal weight	83.6
Overweight	9.8
**Fetal presentation, (%)**	
Cephalic	94.2
Breech or other	5.8
**Birth place, (%)**	
Hospital	99.5
Birth center	0.1
Residence	0.3
Clinic	0.1

Abbreviations: TOLAC, trial of labour after caesarean; BMI, body mass index.

a Overall included women with TOLAC utilisation and planned caesarean delivery.

Table 2 shows that the adjusted prevalence ratios (aPRs) of unsuccessful TOLAC in Hispanic women were higher than non-Hispanic women (1.02, 1.01–1.04; 1.31, 1.26–1.35). Higher BMI, higher gestation weight gain, maternal smoking, and more than one previous caesarean were also positively associated with unsuccessful TOLAC. Regarding maternal comorbidities, majorities increased risk to suffer from the unsuccessful TOLAC. Compared with cephalic, the aPRs of unsuccessful TOLAC were 2.13 (2.12–2.13) for breech or other fetal presentation. In propensity score stratification analysis, TOLAC utilisation was associated with a significantly lower risk of maternal unplanned hysterectomy, admission to ICU, and surfactant (adjusted odds ratios [aORs]: 0.60, 0.60–0.61; 0.84, 0.84–0.85; 0.80, 0.74–0.84; Table 3).

**Table 2:** Sample characteristics associated with unsuccessful TOLAC and TOLAC utilisation during 2012–2020 in the US.

Characteristics	Unsuccessful TOLAC (*N*=245,981)		TOLAC utilisation (*N*=867,843)	
	%	Adjusted PRs^d^ (95% CIs)	%	Adjusted PRs^d^ (95% CIs)
**Maternal characteristics**				
**Maternal age**^a^^,^^b^**, years**				
<25	30.1	1	17.3	1
25–34	27.8	0.88 (0.86–0.90)	18.3	1.17 (1.16–1.17)
≥35	28.8	0.87 (0.85–0.89)	16.7	1.13 (1.13–1.14)
P for trend^c^	<.001		<.001	
**Maternal race**^a^^,^^b^				
Non-Hispanic	28.1	1	18.1	1
Hispanic and Latino American	28.2	1.02 (1.01–1.04)	16.9	0.91 (0.90–0.91)
Other Hispanic	35.0	1.31 (1.26–1.35)	14.9	0.85 (0.84–0.86)
**Gestation age**^a^^,^^b^**, weeks**				
<37	30.3	1	17.3	1
37–42	28.0	0.94 (0.93–0.94)	17.5	0.99 (0.98–0.99)
>42	29.3	0.97 (0.96–0.97)	22.1	1.25 (1.24–1.25)
P for trend c	<.001		<.001	
**Maternal BMI**^a^^,^^b^**, kg/m^2^**				
Underweight	22.0	1	21.4	1
Normal weight	24.1	1.13 (1.12–1.14)	21.0	0.99 (0.97–0.99)
Overweight	28.6	1.32 (1.31–1.33)	18.3	0.88 (0.87–0.89)
Obesity	32.2	1.59 (1.58–1.60)	15.6	0.68 (0.67–0.70)
P for trend c	<.001		<.001	
**Gestation weight gain**^a^^,^^b^**, pounds**				
<20	28.1	1	17.6	1
20–40	27.5	1.10 (1.08–1.11)	18.3	0.92 (0.91–0.93)
>40	32.2	1.28 (1.27–1.29)	15.8	0.79 (0.78–0.80)
P for trend^c^	<.001		<.001	
**Maternal smoking**^a^^,^^b^				
No	28.2	1	18.0	1
Yes	30.1	1.06 (1.05–1.06)	14.6	0.77 (0.76–0.79)
**Prenatal care beginning**^a^^,^^b^**, month**				
0	26.7	1	23.7	1
1^st^–3^r^^d^	28.8	1.03 (1.03–1.04)	16.9	0.71 (0.69–0.73)
4^th^–6^th^	27.3	0.95 (0.94–0.96)	19.3	0.84 (0.82–0.85)
After 7^th^	27.8	0.96 (0.91–1.01)	20.7	0.91 (0.90–0.94)
P for trend c	<.001		<.001	
**Number of previous caesarean**^a^^,^^b^				
<2	27.8	1	18.8	1
≥2	51.9	1.72 (1.72–1.73)	5.0	0.26 (0.25–0.27)
**Maternal co-morbidities**				
Prepregnancy diabetes^a^^,^^b^	43.9	1.34 (1.33–1.35)	11.7	0.72 (0.70–0.75)
Gestational diabetes^a^^,^^b^	32.7	1.08 (1.07–1.09)	14.8	0.90 (0.89–0.91)
Prepregnancy hypertensive disorders a^,^^b^	39.6	1.21 (1.20–1.22)	12.5	0.78 (0.76–0.79)
Gestational hypertensive disorders a^,^^b^	38.2	1.25 (1.24–1.26)	14.6	0.86 (0.85–0.88)
Eclampsia a^,^^b^	40.4	1.30 (1.28–1.31)	14.7	0.93 (0.86–1.01)
Preterm a^,^^b^	22.2	0.72 (0.71–0.73)	23.8	1.50 (1.48–1.51)
Gonorrhea b	28.6	-	19.5	1.08 (1.07–1.09)
Syphilis a^,^^b^	27.5	0.86 (0.85–0.88)	19.2	1.14 (1.13–1.15)
Hepatitis B b	29.2	-	22.2	1.05 (1.03–1.06)
**Neonatal characteristics**				
**Birth weight**^a^**, grams**				
Underweight	28.2	1	17.7	-
Normal weight	38.4	1.00 (0.97–1.03)	17.7	-
Overweight	28.0	0.99 (0.99–0.99)	17.8	-
P for trend c	.849		-	
**Fetal presentation**^a^^,^^b^				
Cephalic	27.4	1	18.4	1
Breech or other	59.7	2.13 (2.12–2.13)	8.3	0.46 (0.45–0.47)

Abbreviations: PR, prevalence ratio; CIs, confidence intervals; TOLAC, trial of labour after caesarean; BMI, body mass index.

a Chi-sq *P*<0.05 for association with unsuccessful TOLAC.

b Chi-sq *P*<0.05 for association with TOLAC utilisation.

c P for linear trend from maternal age, gestation age, maternal BMI, gestation weight gain, prenatal care beginning, birth weight. No further entering into regression model if Chi-sq showed no association, ‘-‘ was added as regression result.

d Mutually Adjusted for birth place clustering and all maternal and neonatal characteristics in Table 2.

**Table 3:** Association of maternal/neonatal morbidity with TOLAC utilisation and planned caesarean delivery in 2012–2020 in the US.

Outcomes	Overall sample				Overall sample	Propensity score stratification sample
	*N*	Planned caesarean delivery (%)	TOLAC utilisation (%)	*P* value*	Adjusted ORs^c^ (95% CIs)	Adjusted ORs^c^ (95% CIs)
**Maternal morbidity**						
Any maternal morbidity a	52,233	0.83	2.2	<.001	2.61 (2.60–2.62)	2.50 (2.50–2.51)
Maternal transfusion	26,140	0.53	0.55	.069	-	-
Perineal laceration	11,292	0.01	1.3	<.001	141.23 (140.75–141.67)	150.97 (148.24–151.67)
Ruptured uterus	5352	0.07	0.28	<.001	3.66 (3.60–3.72)	3.41 (3.39–3.44)
Unplanned hysterectomy	6574	0.14	0.08	<.001	0.56 (0.55–0.57)	0.60 (0.60–0.61)
Admission to ICU	12,683	0.27	0.21	<.001	0.77 (0.76–0.78)	0.84 (0.84–0.85)
**Neonatal morbidity**						
Any neonatal morbidity b	559,557	11.3	11.3	.673	-	-
Assisted ventilation	234,652	4.8	4.7	<.001	0.97 (0.95–0.98)	0.99 (0.98–0.99)
Admission to NICU	464,507	9.5	9.1	<.001	0.94 (0.93–0.95)	0.95 (0.94–0.95)
Antibiotic therapy	99,209	1.9	2.8	<.001	1.50 (1.49–1.51)	1.55 (1.41–1.65)
Seizures	1571	0.03	0.05	<.001	1.75 (1.74–1.77)	1.84 (1.68–1.95)
Surfactant	25,461	0.52	0.45	<.001	0.80 (0.79–0.81)	0.80 (0.74–0.84)

Abbreviations: OR, odds ratio; CIs, confidence intervals; TOLAC, trial of labour after caesarean; ICU, intensive care unit; NICU, neonatal intensive care unit.

a Any maternal morbidity: women had any of the maternal outcomes (i.e. maternal transfusion, perineal laceration, ruptured uterus, unplanned hysterectomy, admission to intensive care.

b Any neonatal morbidity: infants had any of the neonatal outcomes (i.e. assisted ventilation, admission to neonatal intensive care, antibiotic therapy, seizures, surfactant).

⁎ P value represented Chi-sq associations.

c Adjusted for birth place clustering maternal age, maternal race, maternal BMI, gestational weight gain, smoking, prenatal care beginning, pre-preganancy diabetes, gestational diabetes, pre-pregnacy hypertensive disorders, gestational hypertensive disorders, and preterm, which demonstrated in DAG in Figure S2.

**Figure 1:**
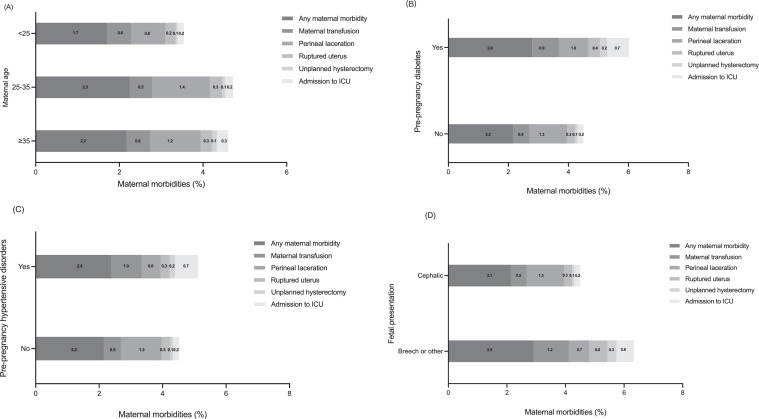
**Prevalence of maternal morbidities among women with specific characteristics who utilised TOLAC during 2012-2020 in the USA**. (A) Maternal age; (B) Pre-pregnancy diabetes; (C) Pre-pregnancy hypertensive disorders; (D) Fetal presentation. ICU, intensive care unit.

Specific maternal and neonatal characteristics associated with maternal morbidity in women who had TOLAC utilization are shown in Table 4 and Figure 1. Table 4 shows women with pre-pregnancy diabetes experienced more maternal complications, for any maternal morbidity (aORs: 1.54, 1.52–1.57), transfusion (aORs: 1.25, 1.22–1.27), laceration (aORs: 1.10, 1.09–1.12), rupture (aORs: 1.45, 1.39–1.53), hysterectomy (aORs: 2.02, 2.00–2.06), and admission to ICU (aORs: 1.99, 1.91–2.06). Besides this, advanced maternal age, pre-pregnancy hypertensive disorders, and birth malpresentation were also identified as major factors associated with most of maternal morbidity in TOLAC women. Figure 1 (Panel A to D) shows detailed percentages of maternal morbidities by maternal age, pre-pregnancy diabetes, pre-pregnancy hypertensive disorders, and birth malpresentation.

**Table 4:** Associations of specific maternal/neonatal characteristics with maternal morbidities among women with TOLAC utilisation in 2012–2020 in the US.

	Any maternal morbidity	Maternal transfusion	Perineal laceration	Ruptured uterus	Unplanned hysterectomy	Admission to ICU
	Adjusted ORs^h^ (95% CIs)	Adjusted ORs^h^ (95% CIs)	Adjusted ORs^h^ (95% CIs)	Adjusted ORs^h^ (95%CIs)	Adjusted ORs^h^ (95% CIs)	Adjusted ORs^h^ (95%CIs)
**Maternal age, years**^a^^,^^c^^,^^d^^,^^e^^,^^f^ (Reference: <25)						
25–34	1.27 (1.26–1.29)	-	1.61 (1.60–1.62)	1.15 (1.13–1.16)	1.75 (1.73–1.77)	1.23 (1.20–1.26)
≥35	1.28 (1.27–1.30)	-	1.49 (1.48–1.50)	1.09 (1.06–1.12)	2.49 (2.45–2.54)	1.62 (1.58–1.68)
*P* for trend^g^	<.001	-	<.001	.001	<.001	<.001
**Maternal Race**^a^^,^^c^^,^^d^ (Reference: Non-Hispanic)						
Hispanic and Latino American	0.70 (0.69–0.72)	-	0.62 (0.61–0.63)	0.70 (0.69–0.72)	-	-
Other Hispanic	0.62 (0.61–0.64)	-	0.55 (0.54–0.55)	0.74 (0.71–0.76)	-	-
**Gestational age, weeks**^a^^,^^b^^,^^c^^d^^.^^e^^,^^f^ (Reference: <37)						
37–42	1.04 (1.03–1.05)	0.84 (0.83–0.84)	2.49 (2.48–2.50)	1.40 (1.32–1.50)	0.53 (0.52-0.54)	0.43 (0.42–0.44)
>42	1.06 (1.05–1.07)	0.92 (0.89–0.94)	2.36 (2.35–2.37)	1.79 (1.64–1.95)	0.59 (0.57-0.59)	0.54 (0.53–0.55)
*P* for trend g	<.001	.031	<.001	<.001	.001	<.001
**Body mass index, kg/m^2^**^c^^,^^f^ (Reference: Under)						
Normal	-	-	1.05 (1.04–1.06)	-	-	0.98 (0.97–0.99)
Over	-	-	0.85 (0.84–0.86)	-	-	0.99 (0.97–1.00)
Obesity	-	-	0.53 (0.52–0.53)	-	-	1.05 (1.04–1.06)
*P* for trend^g^	-	-	<.001	-	-	<.001
**Prenatal care beginning, months**^a^^,^^b^^,^^c^^,^^f^ (Reference: 0)						
1^st^-3^r^^d^	1.16 (1.14–1.17)	0.74 (0.70–0.79)	1.57 (1.55–1.58)	-	-	0.62 (0.61–0.63)
4^t^^h^-6^t^^h^	0.98 (0.97–0.99)	0.85 (0.81–0.87)	1.06 (1.05–1.07)	-	-	0.72 (0.71–0.73)
After 7^t^^h^	0.99 (0.96–1.00)	0.82 (0.74–0.84)	1.17 (1.16–1.18)	-	-	0.83 (0.81–0.86)
*P* for trend^g^	<.001	.020	<.001	-	-	<.001
**Previous caesarean**^b^^,^^c^^,^^e^^,^^f^ (Reference: ≤ 2)						
>2	-	1.56 (1.52–1.60)	0.31 (0.30–0.32)	-	3.09 (3.03–3.16)	1.76 (1.72–1.80)
**Pre-pregnancy diabetes**^a^^,^^b^^,^^c^^d^^.^^e^^,^^f^ (Reference: No)						
Yes	1.54 (1.52–1.57)	1.25 (1.22–1.27)	1.10 (1.09–1.12)	1.45 (1.39–1.53)	2.02 (2.00–2.06)	1.99 (1.91–2.06)
**Gestation diabetes**^a^^,^^b^^,^^f^ (Reference: No)						
Yes	1.14 (1.12–1.15)	1.07 (1.05–1.08)	-	-	-	1.12 (1.08–1.17)
**Pre-pregnancy hypertensive disorders**^a^^,^^b^^,^^c^^d^^.^^e^^,^^f^ (Reference: No)						
Yes	1.05 (1.04–1.06)	1.55 (1.52–1.56)	0.64 (0.63–0.65)	1.01 (0.98–1.04)	1.46 (1.43–1.50)	2.71 (2.68–2.72)
**Gestation hypertensive disorders**^a^^,^^b^^,^^c^^d^^.^^e^^,^^f^ (Reference: No)						
Yes	1.32 (1.29–1.35)	2.03 (2.01–2.05)	0.83 (0.82–0.84)	1.38 (1.36–1.40)	1.39 (1.37–1.41)	2.38 (2.35–2.41)
**Preterm**^a^^,^^b^^,^^c^^.^^e^^,^^f^ (Reference: No)						
Yes	1.03 (0.97–1.04)	1.36 (1.35–1.37)	0.73 (0.72–0.74)	-	1.14 (1.11–1.18)	1.41 (1.37–1.46)
**Syphilis**^b^^,^^c^^d^^.^^e^^,^^f^ (Reference: No)						
Yes	-	1.68 (1.63–1.71)	1.03 (1.02–1.04)	0.91 (0.87–0.94)	3.56 (3.43–3.70)	1.67 (1.60–1.74)
**Birth weight, grams** (Reference: Under)						
Under	-	-	-	-	-	-
Normal	-	-	-	-	-	-
Over	-	-	-	-	-	-
P for trend^g^	-	-	-	-	-	-
**Fetal presentation**^a^^,^^b^^,^^c^^d^^.^^e^^,^^f^ (Reference: Cephalic)						
Breech/other	1.32 (1.31–1.35)	1.56 (1.52–1.58)	0.79 (0.78–0.80)	2.50 (2.46–2.55)	3.16 (3.03–3.28)	2.30 (2.28–2.32)

Abbreviations: OR, odds ratio; CI, confidence interval; TOLAC, trial of labour after caesarean; ICU, intensive care unit.

a Chi-sq *P*<0.05 for association with any neonatal morbidity.

b Chi-sq *P*<0.05 for association with assisted ventilation.

c Chi-sq *P*<0.05 for association with admit to NICU.

d Chi-sq *P*<0.05 for association with surfactant.

e Chi-sq *P*<0.05 for association with antibiotic therapy.

f Chi-sq *P*<0.05 for association with seizures. No further entering into regression model if Chi-sq showed no association, ‘-‘ was added as regression result.

g P for linear trend from maternal age, gestation age, maternal BMI, prenatal care beginning, birth weight.

h Mutually Adjusted for state and hospital clustering and all maternal and neonatal characteristics in Table 4.

**Figure 2:**
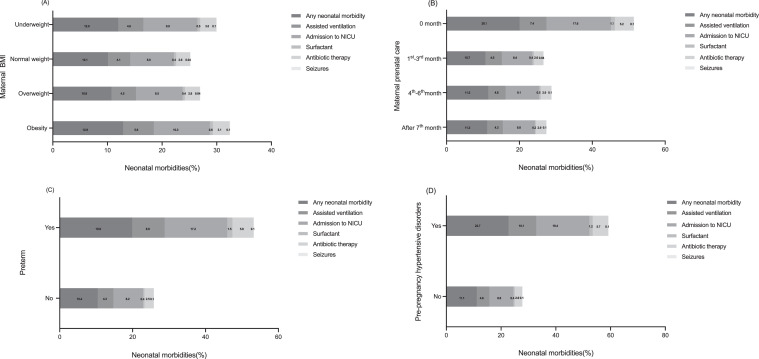
**Prevalence of neonatal morbidities among women with specific characteristics who utilised TOLAC during 2012-2020 in the USA**. (A) Maternal BMI; (B) Maternal prenatal care; (C) Preterm; (D) Pre-pregnancy hypertensive disorders. BMI, body mass index; NICU, neonatal intensive care unit.

Table 5 and Figure 2 show neonatal morbidity rates and associations with specific maternal and neonatal characteristics in TOLAC women. Obesity, pre-pregnancy hypertensive disorders, and preterm birth were positively associated with all neonatal morbidities. Preterm had an increased likelihood of any morbidity (aORs: 1.46, 1.45–1.46), ventilation (aORs: 1.62, 1.61–1.62), NICU admission (aORs: 1.52, 1.50–1.52), surfactant (aORs: 2.14, 2.10–2.17), antibiotic therapy (aORs: 1.65, 1.63–1.67), and seizures (aORs: 1.34, 1.33–1.35). However, women who started prenatal care at the first to the third month had lower aORs in experiencing all neonatal morbidities (any morbidity: 0.70, 0.69–0.70; ventilation: 0.85, 0.84–0.86; NICU admission: 0.64, 0.63–0.64; surfactant: 0.76, 0.74–0.77; antibiotic therapy: 0.77, 0.76–0.78; seizures: 0.63, 0.62–0.64). Figure 2 (Panel A to D) shows detailed percentages of neonatal morbidities by maternal BMI, prenatal care beginning, pre-pregnancy hypertensive disorders, and preterm birth.

**Table 5:** Associations of specific maternal/neonatal characteristics with neonatal morbidities among women with TOLAC utilisation in 2012–2020 in the US.

	Any neonatal morbidity	Assisted ventilation	Admission to NICU	Surfactant	Antibiotic therapy	Seizures
	Adjusted ORs^h^ (95% CIs)	Adjusted ORs^h^ (95% CIs)	Adjusted ORs^h^ (95% CIs)	Adjusted ORs^h^ (95% CIs)	Adjusted ORs^h^ (95% CIs)	Adjusted ORs^h^ (95% CIs)
**Maternal age**^a^^,^^b^^,^^c^^,^^d^^.^^e^ (Reference: <25)						
25–34	0.97 (0.96–0.98)	1.01 (1.00–1.01)	0.98 (0.97–0.98)	0.93 (0.91–0.95)	0.93 (0.92–0.93)	-
≥35	1.02 (1.00–1.03)	1.02 (1.02–1.03)	0.99 (0.98–0.99)	0.87 (0.86–0.89)	0.87 (0.86–0.88)	-
*P* for trend	<.001	.560	<.001	<.001	<.001	-
**Maternal Race**^a^^,^^b^^,^^c^^d^^,^^f^ (Reference: Non-Hispanic)						
Hispanic and LatinoAmerican	0.90 (0.90–0.91)	0.73 (0.72–0.73)	0.95 (0.95–0.96)	0.68 (0.66–0.71)	-	0.53 (0.52–0.54)
Other Hispanic	1.02 (1.01–1.03)	0.79 (0.78–0.80)	1.13 (1.12–1.14)	0.49 (0.48–0.40)	-	0.24 (0.23–0.25)
**Gestational age, weeks**^a^^,^^b^^,^^c^^d^^.^^e^^,^^f^ (Reference: <37)						
37–42	0.13 (0.13–0.14)	0.20 (0.20–0.21)	0.10 (0.10–0.11)	0.03 (0.03–0.04)	0.17 (0.16–0.17)	0.67 (0.66–0.68)
>42	0.15 (0.14–0.16)	0.25 (0.24–0.25)	0.11 (0.10–0.12)	0.06 (0.05–0.06)	0.19 (0.18–0.19)	0.70 (0.69–0.70)
*P* for trend	<.001	<.001	<.001	<.001	<.001	<.001
**Body mass index, kg/m^2^**^a^^,^^b^^,^^c^^,^^d^^,^^e^^,^^f^ (Reference: Under)						
Normal	1.01 (1.00–1.02)	1.02 (1.01–1.04)	0.99 (0.97–1.01)	0.98 (0.96–1.01)	1.16 (1.15–1.17)	0.89 (0.89–0.90)
Over	1.05 (1.04–1.06)	1.12 (1.10–1.14)	1.02 (1.01–1.02)	1.12 (1.11–1.13)	1.17 (1.16–1.17)	0.84 (0.81–0.87)
Obesity	1.15 (1.14–1.16)	1.29 (1.25–1.33)	1.10 (1.09–1.12)	1.30 (1.29–1.31)	1.28 (1.27–1.29)	1.02 (1.01–1.05)
*P* for trend	<.001	<.001	<.001	<.001	<.001	<.001
**Prenatal care beginning, months**^a^^,^^b^^,^^c^^,^^d^^,^^e^^,^^f^ (Reference: 0)						
1^st^-3^r^^d^	0.70 (0.69–0.70)	0.85 (0.84–0.86)	0.64 (0.63–0.64)	0.76 (0.74–0.77)	0.77 (0.76–0.78)	0.63 (0.62–0.64)
4^t^^h^-6^t^^h^	0.71 (0.70–0.72)	0.89 (0.88–0.91)	0.64 (0.62–0.64)	0.66 (0.65–0.67)	0.80 (0.79–0.81)	0.79 (0.78–0.80)
After 7^t^^h^	0.71 (0.69–0.73)	0.86 (0.84–0.87)	0.68 (0.67–0.69)	0.44 (0.43–0.45)	0.83 (0.82–0.84)	0.77 (0.76–0.77)
*P* for trend	<.001	.173	<.001	.010	<.001	<.001
**Previous caesarean**^a^^,^^b^^,^^c^^d^^.^^e^^,^^f^ (Reference: ≤ 2)						
> 2	1.13 (1.05–1.22)	1.25 (1.23–1.26)	1.24 (1.23–1.25)	1.44 (1.41–1.48)	0.97 (0.96–1.05)	1.64 (1.63–1.65)
**Pre-pregnancy diabetes**^a^^,^^b^^,^^c^^d^^.^^e^^,^^f^ (Reference: No)						
Yes	2.56 (2.50–2.61)	1.92 (1.89–1.96)	2.83 (2.76–2.89)	1.51 (1.46–1.56)	1.76 (1.71–1.81)	1.44 (0.77–2.68)
**Gestation diabetes**^a^^,^^b^^,^^c^^,^^e^ (Reference: No)						
Yes	1.36 (1.34–1.37)	1.20 (1.19–1.21)	1.40 (1.39–1.40)	-	1.27 (1.26–1.28)	-
**Pre-pregnancy hypertensive disorders**^a^^,^^b^^,^^c^^d^^.^^e^^,^^f^ (Reference: No)						
Yes	1.46 (1.45–1.47)	1.43 (1.41–1.44)	1.43 (1.42–1.44)	1.14 (1.09–1.20)	1.24 (1.23–1.25)	1.94 (1.92–1.95)
**Gestation hypertensive disorders**^a^^,^^b^^,^^c^^d^^.^^e^^,^^f^ (Reference: No)						
Yes	1.51 (1.50–1.51)	1.41 (1.40–1.43)	1.52 (1.51–1.53)	0.89 (0.88–0.90)	1.16 (1.15–1.17)	1.59 (1.58–1.61)
**Preterm**^a^^,^^b^^,^^c^^.^^d^^,^^e^^,^^f^ (Reference: No)						
Yes	1.46 (1.45–1.46)	1.62 (1.61–1.62)	1.52 (1.50–1.52)	2.14 (2.10–2.17)	1.65 (1.63–1.67)	1.34 (1.33–1.35)
**Syphilis**^a^^,^^b^^,^^c^^.^^e^ (Reference: No)						
Yes	2.53 (2.43–2.63)	1.41 (1.39–1.43)	2.64 (2.50–2.77)	-	3.01 (2.95–3.07)	-
**Birth weight, grams** (Reference: Under)						
Under	-	-	-	-	-	-
Normal	-	-	-	-	-	-
Over	-	-	-	-	-	-
P for trend^c^	-	-	-	-	-	-
**Fetal presentation**^a^^,^^b^^,^^c^^d^^.^^e^^,^^f^ (Reference: Cephalic)						
Breech/other	1.60 (1.59–1.62)	1.91 (1.88–1.95)	1.53 (1.51–1.55)	2.14 (2.09–2.18)	1.20 (1.18–1.21)	2.78 (2.75–2.81)

Abbreviations: OR, odds ratio; CI, confidence interval; TOLAC, trial of labour after caesarean; NICU, neonatal intensive care unit.

a Chi-sq *P*<0.05 for association with any neonatal morbidity.

b Chi-sq *P*<0.05 for association with assisted ventilation.

c Chi-sq *P*<0.05 for association with admit to NICU.

d Chi-sq *P*<0.05 for association with surfactant.

e Chi-sq *P*<0.05 for association with antibiotic therapy.

f Chi-sq *P*<0.05 for association with seizures. No further entering into regression model if Chi-sq showed no association, ‘-‘ was added as regression result. ^g^P for linear trend from maternal age, gestation age, maternal BMI, prenatal care beginning, birth weight.

h Mutually Adjusted for state and hospital clustering and all maternal and neonatal characteristics in Table 5.

Table S3 and Table S4 showed comparisons of maternal and neonatal morbidities between only TOLAC utilisation and TOLAC with induction, between women who did not have previous caesarean and had the previous caesarean with unplanned caesarean deliveries.

## Discussion

The current study revealed four findings as follows. First, we found that 17.7% of women with history of at least one prior caesarean delivery attempted TOLAC in the US during the period 2012–2020, of which 28.3% experienced unsuccessful TOLAC. Second, several maternal and/or neonatal characteristics such as maternal race, maternal body mass index, gestational weight gain, more than 2 previous caesarean deliveries, maternal co-morbidities, and fetal presentation, were significantly associated with TOLAC utilisation and unsuccessful TOLAC. Third, according to propensity score stratification analysis, there were significant differences in the risk of individual maternal and/or neonatal morbidity between women with TOLAC utilisation and women who had planned caesarean deliveries. Fourth, we also observed that women with specific maternal and/or neonatal characteristics (i.e., maternal age, maternal race, gestational age, maternal body mass index, prenatal care, number of prior caesarean deliveries, maternal co-morbidities, birth weight, fetal presentation) had an increased risk of individual maternal and neonatal morbidity.

Considering the rates of women who attempted TOLAC from the 1980s to the 2020s in the US, prior studies have demonstrated that 3%–62% of pregnant women attempted TOLAC. A study in 1996 reported that approximately 44% of women had TOLAC in comparison with 62% in a study started before 1996.^25^, ^26^, ^27^ Nevertheless, TOLAC utilisation rates in 2018 was reported at 28% and in 2020 at 3%, respectively.^18^^,^^28^ The reduced TOLAC utilisation rate might reflect a national trend during recent years. A less TOLAC-friendly environment and inadequate prenatal care might have lowered the threshold for caesarean delivery in recent years.

In this study, we observed women with TOLAC utilisation and unsuccessful TOLAC had inverse relationships in Hispanic ethnicity, overweight or obesity women, more than 20 pounds weight gain, having at least 2 previous caesarean deliveries, malpresentation, and maternal co-morbidities. Our findings are in line with previous studies, Dombrowski et al conducted a retrospective cohort study to determine older maternal age, preterm birth, maternal obesity, and some maternal diseases were significantly associated with a lower likelihood of TOLAC utilisation.^18^ Thapsamuthdechakorn et al also analysed secondary data to show high maternal body mass index and malpresentation were significantly associated with a higher risk of unsuccessful TOLAC.^28^ Another trial in 536 pregnant women by Herman et al showed maternal age (OR 0.95, 95% CI 0.91–0.99, *p* = .04), maternal body mass index (OR 0.92, 95% CI 0.88–0.97, *p* = .002), and diabetes mellitus (OR 0.25, 95% CI 0.07–0.94, *p* = .04) were associated with TOLAC utilisation.^29^ Therefore, women without term pregnancies, with obesity, experiencing more than 2 previous caesarean deliveries, having maternal co-morbidities (i.e., diabetes, hypertensive disorders) and non-vertex presentation would not be considered as the appropriate candidates for TOLAC.

We further found that women who chose TOLAC were more likely to have individual maternal and neonatal morbidity (maternal morbidity, 0.08–2.20%; neonatal morbidity, 0.05–11.30%) compared with women who chose planned caesarean deliveries (maternal morbidity, 0.01–0.83%; neonatal morbidity, 0.03–11.30%). This may due to the probability of maternal morbidity was higher with unsuccessful TOLAC (i.e. unplanned caesarean), and increased the risks of surgical wounds, hemorrhagic complications requiring hysterectomy or blood transfusion, and infectious complications.^30^^,^^31^ At the same time, in Table S6, we observed women with previous caesarean deliveries who experienced unplanned caesarean increased risks of perineal laceration, ruptured uterus, unplanned hysterectomy, and neonatal surfactant (1.94, 1.25–2.63; 15.50, 15.50–15.51; 2.05, 1.98–2.09; 1.14, 1.14–1.14). This could be supported by our result of having previous caesarean, particularly more than 2 times gained 72% more likely to experience unsuccessful TOLAC, even resulted in higher chance of maternal morbidities. Therefore, TOLAC should be considered carefully for pregnant women, particularly women with certain factors related to previous caesarean birth or other factors that would be contraindications to receiving TOLAC.

In this study, we determined that non-Hispanic women who were obese, over age 25 years, with gestational age more than 42 weeks, and who had more than 2 previous caesarean deliveries, suffered from pre-pregnancy diabetes and pre-pregnancy hypertensive disorders, and fetal malpresentation might be less appropriate candidates for TOLAC because they had higher odds of maternal morbidities in our study. Women with non-Hispanic ethnicity, less than 37-week gestation age, obesity, without prenatal care, less than 1-year pregnancy interval, more than 2 previous caesarean deliveries, experienced pre-pregnancy diabetes/hypertensive disorders, preterm births, syphilis, and fetal malpresentation might be less appropriate candidates for TOLAC because they had higher odds of neonatal morbidities. Consistent with previous studies: Cheng et al showed that the prevalence of hysterectomy and placenta previa in the fourth caesarean delivery reach 2.4% and 2.1% in comparison with the third caesarean delivery at 0.9% and 1.1%^32^; another study by Macones et al conducted found the risk of uterine rupture increased from 0.9% to 1.8% in TOLAC women with 1 versus 2 previous caesarean deliveries^33^; an increased risk of uterine rupture more than 40-week gestation age was observed by Kiran et al.^34^

According to the SOGC Guidelines for Vaginal Trial Delivery after Caesarean Section (2019 Edition),^35^ physicians and obstetricians should assess the application of TOLAC for pregnant women by reviewing previous maternal medical records on the number of caesarean deliveries, previous diseases, body mass index, and location and type of uterine incision, among others. It is important to consider the balance between the benefits from TOLAC utilisation and risks of unsuccessful TOLAC. Hence, health care providers need to clarify the indications for TOLAC utilisation among patients and the decision for TOLAC use should be made jointly by health care providers and patients. Informed consent should be provided to all patients who agree to attempt TOLAC following adequate obstetrician–patient communication. Given the possibility of severe maternal and/or neonatal morbidities owing to unsuccessful TOLAC, a plan after unsuccessful TOLAC should be developed. Most importantly, maternal/neonatal outcomes should be optimised, which requires the use of TOLAC in hospitals offering emergent caesarean delivery so as to reduce the time from the decision for surgery to delivery of the fetus and reduce the risk of maternal and/or neonatal morbidities.

This study has several strengths, including directly addressing the ultimate health outcomes of the births; the large sample size, which allowed for cross-stratification according to TOLAC utilisation and planned caesarean deliveries; and using data from multiple US states, making the findings more generalisable. Moreover, propensity score stratification was used to more rigorously determine differences in maternal and neonatal morbidity between women with TOLAC utilisation and those with planned caesarean deliveries, conceptualising as a set of quasi-randomised controlled trials. We identified individual maternal and/or neonatal morbidity instead of using a composite variable of any adverse maternal and/or neonatal outcomes, given the large disparities in prevalence.

This study also has several limitations. First, we conducted a retrospective analysis in this study. Although we applied a rigorous algorithm to determine TOLAC utilisation by integrating data from both the birth certificate and maternal medical records, this information might not accurately capture the intended mode of delivery and we might have underestimated the TOLAC occurrence. Second, we did not collect information related to closure of uterine incision, episiotomy, stillbirth, length of hospital stay, admission to neonatal ITU, alcohol consumption, specific states and hospital of sample enrollment, and postnatal admission due to no such data provided in this dataset.

Our study findings showed that TOLAC utilisation was associated with a modest rise in maternal and/or neonatal morbidity when comparing with the planned caesarean delivery. When utilising TOLAC, specific maternal and neonatal factors in pregnant women should be considered in conjunction with the potential benefits of TOLAC in preventing caesarean delivery in subsequent pregnancies.

## Contributors

Yinzi Jin & Hanxu Shi were responsible for study conceptualisation. Hanxu Shi & Siwen Li were responsible for data curation. Hanxu Shi, Siwen Li, Jin Lv & Harry H.X. Wang were responsible for formal analysis. Yinzi Jin was responsible for funding acquisition. Hanxu Shi, Siwen Li, Jin Lv & Harry H.X. Wang were responsible for investigation and methodology.

Yinzi Jin was responsible for supervision. Hanxu Shi, Siwen Li, Jin Lv, Harry H.X. Wang & Qingxiang Hou were responsible for verifying the underlying data. Hanxu Shi & Siwen Li were responsible for original draft the manuscript. Yinzi Jin, Jin Lv & Harry H.X. Wang were responsible for reviewing and editing the manuscript. All authors had full access to all the data in the study and accepted responsibility to submit for publication.

## Data sharing statement

All data sharing and collaboration requests should be directed to the corresponding author.

## Declaration of interests

There are no conflicts of interest in this study.

## Appendix Supplementary materials

Image, application 1
