# Supplementary material for: Maternal and neonatal characteristics associated with clinical outcomes of TOLAC from 2012–20 in the USA: Evidence from a retrospective cohort study
Source: eClinicalMedicine. 2022 Sep 28;54:101681. doi: 10.1016/j.eclinm.2022.101681 (PMC9526178; doi:10.1016/j.eclinm.2022.101681)
Supplement: Supplementary file 1 [file mmc1.pdf]

Table S1. Algorithm Used to Identify Trial of Labour

Any one of the following:	
Birth certificate	Method of delivery = vaginal delivery or trial of labour attempted
ICD-9 procedure code on maternal hospital discharge record	73.01,73.1, 73.4

Figure S1. Flow diagram of inclusion and exclusion criteria of the sample in main analyses

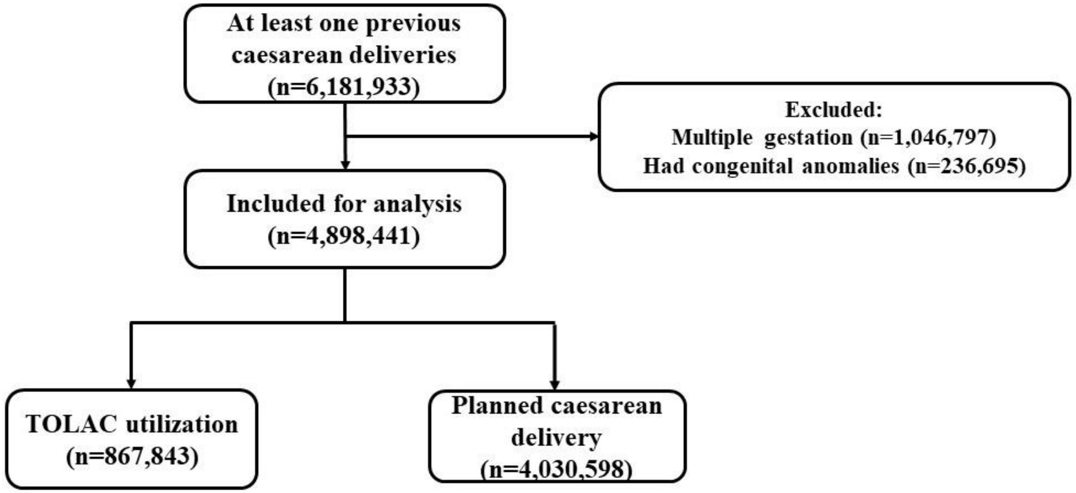

Abbreviations: TOLAC, trial of labour after caesarean

Figure S2. Identification of adjusted covariates

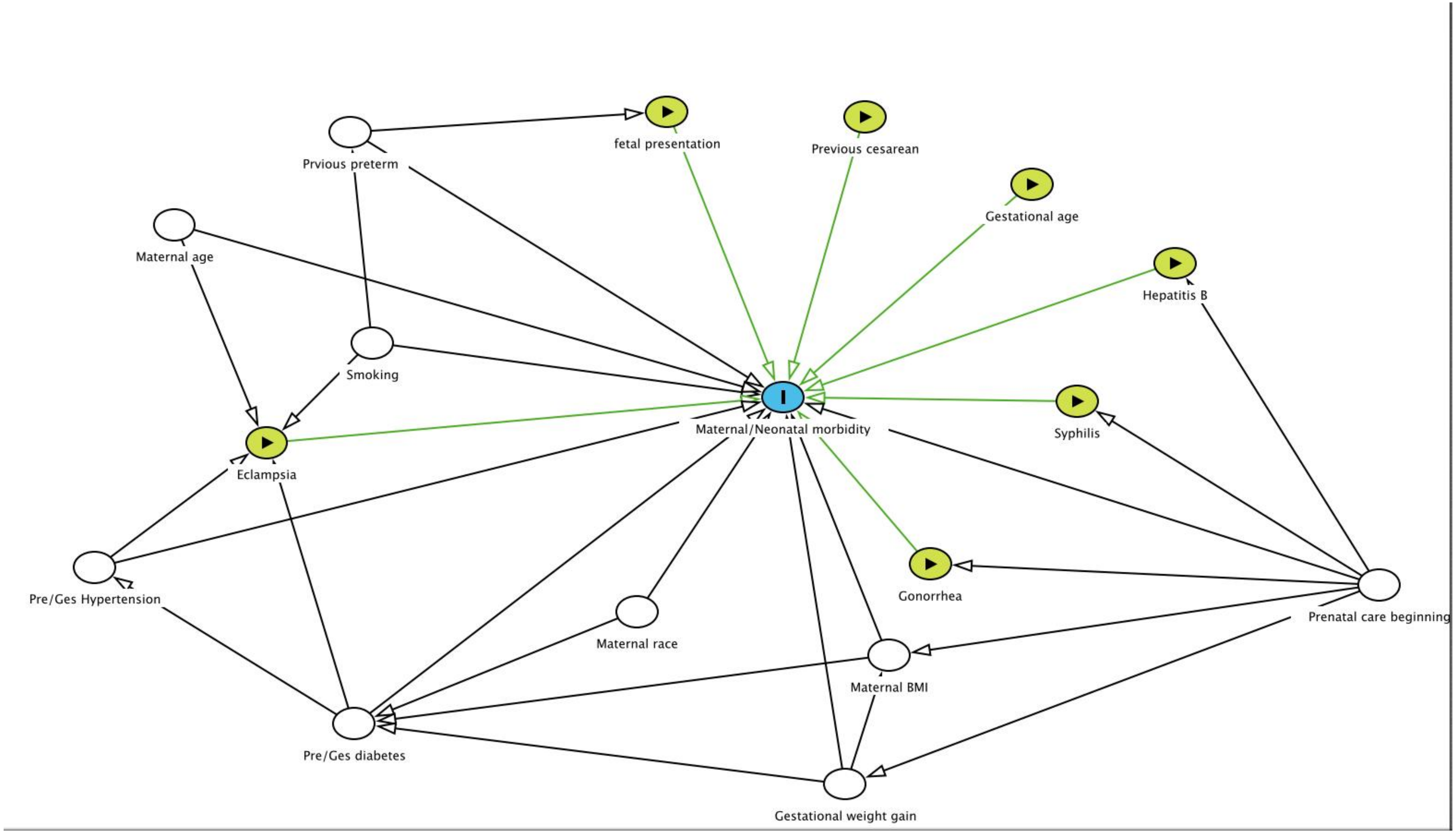

Green nodes represent exposure; blue nodes represent outcome of interest; white nodes represent adjusted variables

Table S2. Description of missing data (N=4,898,441)

	Information missing		Information available	
**Total subjects included in main analyses**	N	%	N	%
			4,898,441	100.0
**Outcomes**				
Maternal transfusion	5,682	0.1	4,892,759	99.9
Perineal laceration	5,682	0.1	4,892,759	99.9
Ruptured uterus	5,682	0.1	4,892,759	99.9
Unplanned hysterectomy	5,682	0.1	4,892,759	99.9
Admission to ICU	5,682	0.1	4,892,759	99.9
Assisted ventilation	5,273	0.1	4,893,168	99.9
Admission to NICU	5,273	0.1	4,893,168	99.9
Antibiotic therapy	5,273	0.1	4,893,168	99.9
Seizures	5,273	0.1	4,893,168	99.9
Surfactant	5,273	0.1	4,893,168	99.9
**Covariates**				
Maternal age	0	0.0	4,898,441	100.0
Maternal race	38,670	0.7	4,859,771	99.3
Gestation age	2,096	0.04	4,896,345	99.96
Maternal BMI	42,867	0.8	4,855,574	99.2
Gestation weight gain	77,309	1.5	4,821,132	98.5
Maternal smoking	0	0.0	4,898,441	100.0
Prenatal care beginning	0	0.0	4,898,441	100.0
Number of previous caesarean	13,954	0.2	4,884,487	99.8
Composite maternal co-morbidities [a]	0	0.0	4,898,441	100.0
Composite maternal co-morbidities [b]	13,108	0.2	4,885,333	99.8
Birth weight	725	0.01	4,897,716	99.99
Fetal presentation	68,254	1.3	4,830,187	98.7

Abbreviations: BMI, body mass index; ICU, intensive care unit; NICU, neonatal intensive care unit

[a] Missing on the composite maternal co-morbidities included pre-pregnancy Diabetes, gestational Diabetes, pre-pregnancy Hypertensive disorders, gestational Hypertensive disorders, Eclampsia, Preterm.

[b] Missing on the composite maternal co-morbidities included Gonorrhoea Syphilis, Hepatitis B

Table S3. Association of maternal/neonatal morbidity with only TOLAC utilisation and TOLAC with induction in 2012-2020 in the US

Outcomes	Overall sample			Overall sample	Propensity score stratification sample
	Only TOLAC (%)	TOLAC with induction (%)	P value*	Adjusted ORs # (95% CIs)	Adjusted ORs # (95% CIs)
**Maternal morbidity**					
Any maternal morbidity [a]	2.1	2.6	<.001	1.25 (1.24-1.26)	1.27 (1.19-1.36)
Maternal transfusion	0.50	0.73	<.001	1.39 (1.38-1.39)	1.44 (1.29-1.58)
Perineal laceration	1.3	1.3	.233	-	-
Ruptured uterus	0.23	0.47	<.001	2.02 (1.99-2.04)	2.04 (1.97-2.13)
Unplanned hysterectomy	0.08	0.10	.022	1.11 (1.09-1.14)	1.13 (1.12-1.14)
Admission to ICU	0.19	0.26	<.001	1.14 (1.12-1.16)	1.07 (1.07-1.08)
**Neonatal morbidity**					
Any neonatal morbidity [b]	11.4	11.1	.041	0.88 (0.87-0.88)	0.89 (0.85-0.91)
Assisted ventilation	4.7	4.7	.853	-	-
Admission to NICU	9.2	8.6	<.001	0.82 (0.81-0.82)	0.84 (0.80-0.86)
Antibiotic therapy	2.9	2.7	.003	0.88 (0.87-0.88)	0.89 (0.84-0.93)
Seizures	0.05	0.05	.556	-	-
Surfactant	0.50	0.24	<.001	0.41 (0.40-0.41)	0.42 (0.39-0.44)

Abbreviations: ORs, odds ratios; CIs, confidence intervals; TOLAC, trial of labour after caesarean; ICU, intensive care unit; NICU, neonatal intensive care unit

[a] Any maternal morbidity: women had any of the maternal outcomes (i.e. maternal transfusion, perineal laceration, ruptured uterus, unplanned hysterectomy, admission to intensive care

[b] Any neonatal morbidity: infants had any of the neonatal outcomes (i.e. assisted ventilation, admission to neonatal intensive care, antibiotic therapy, seizures, surfactant)

* P value represented Chi-sq associations

#Adjusted for state and hospital clustering maternal age, maternal race, maternal BMI, gestational weight gain, smoking, prenatal care beginning, pre-preganancy diabetes, gestational diabetes, pre-pregnancy hypertensive disorders, gestational hypertensive disorders, and preterm, which demonstrated in DAG in Figure S2.

Table S4. Association of maternal/neonatal morbidity with unplanned caesarean delivery in women without previous caesarean and women with previous caesarean in 2012-2020 in the US

Outcomes	Overall sample			Overall sample	Propensity score stratification sample
	Without previous caesarean (%)	With previous caesarean (%)	P value*	Adjusted ORs # (95% CIs)	Adjusted ORs # (95% CIs)
**Maternal morbidity**					
Any maternal morbidity [a]	1.1	2.0	<.001	1.80 (1.79-1.80)	1.84 (1.70-1.98)
Maternal transfusion	0.79	0.93	<.001	1.15 (1.14-1.15)	1.18 (1.10-1.25)
Perineal laceration	0.03	0.05	<.001	1.61 (1.61-1.62)	1.94 (1.25-2.63)
Ruptured uterus	0.06	0.82	<.001	13.54 (13.53-13.57)	15.50 (15.50-15.51)
Unplanned hysterectomy	0.08	0.18	<.001	1.90 (1.89-1.91)	2.05 (1.98-2.09)
Admission to ICU	0.36	0.41	<.001	1.10 (1.09-1.10)	1.10 (1.05-1.15)
**Neonatal morbidity**					
Any neonatal morbidity [b]	17.1	16.7	<.001	0.90 (0.89-0.90)	0.90 (0.89-0.92)
Assisted ventilation	7.4	7.5	.112	-	-
Admission to NICU	13.0	13.3	<.001	1.01 (0.97-1.03)	0.93 (0.87-1.02)
Antibiotic therapy	4.3	3.9	<.001	0.83 (0.82-0.85)	0.81 (0.78-0.85)
Seizures	0.08	0.09	.224	-	-
Surfactant	0.48	0.62	<.001	1.05 (1.03-1.08)	1.14 (1.14-1.15)

Abbreviations: ORs, odds ratios; CIs, confidence intervals; TOLAC, trial of labour after caesarean; ICU, intensive care unit; NICU, neonatal intensive care unit

[a] Any maternal morbidity: women had any of the maternal outcomes (i.e. maternal transfusion, perineal laceration, ruptured uterus, unplanned hysterectomy, admission to intensive care

[b] Any neonatal morbidity: infants had any of the neonatal outcomes (i.e. assisted ventilation, admission to neonatal intensive care, antibiotic therapy, seizures, surfactant)

* P value represented Chi-sq associations

#Adjusted for state and hospital clustering maternal age, maternal race, maternal BMI, gestational weight gain, smoking, prenatal care beginning, pre-preganancy diabetes, gestational diabetes, pre-pregnancy hypertensive disorders, gestational hypertensive disorders, and preterm, which demonstrated in DAG in Figure S2.

Figure S3. Flow chart of National Vital Statistics System Data

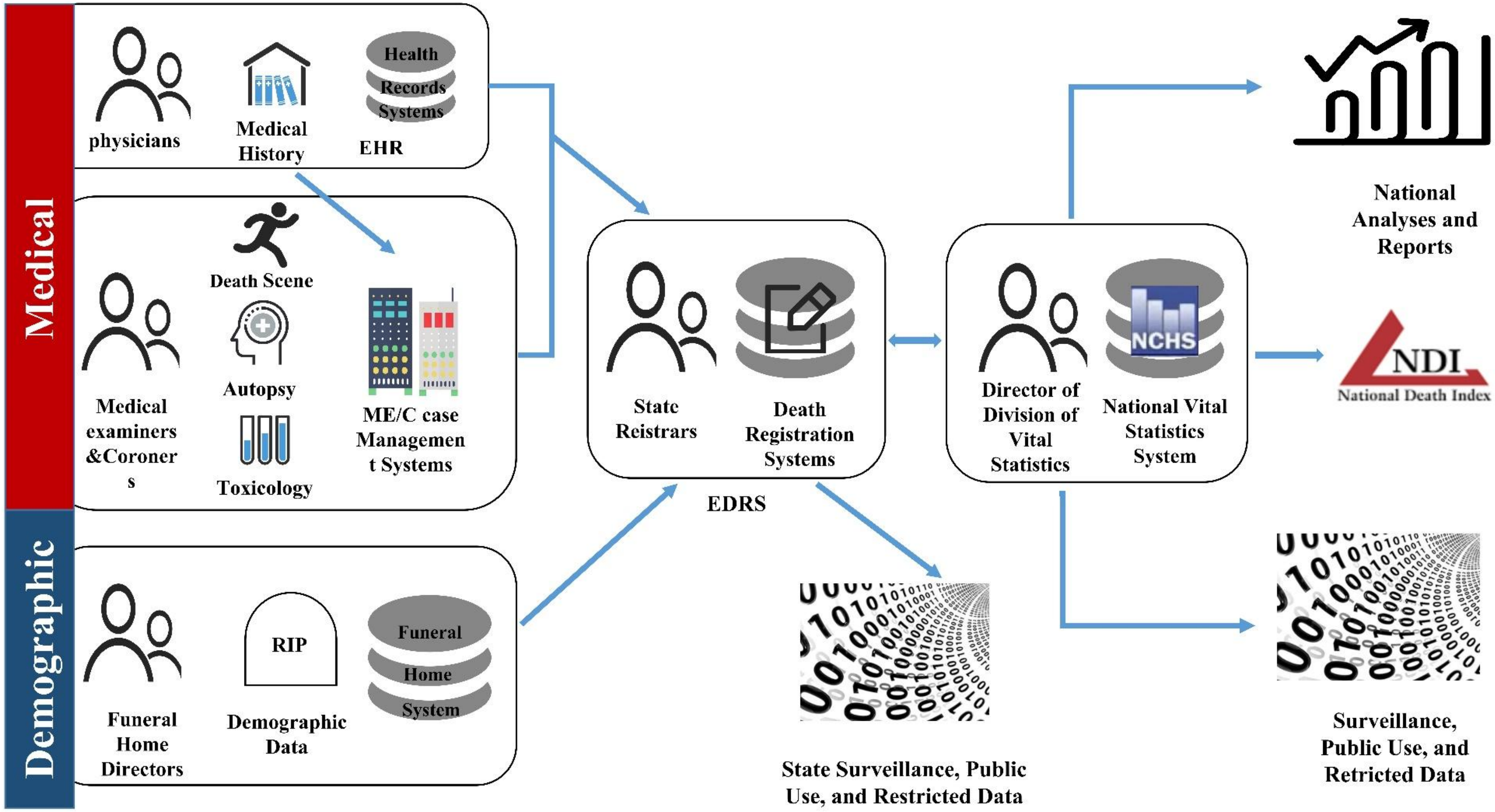

Sutton, P. D. (2021). National Vital Statistics System Data Modernization and NVSS Modernization Community of Practice [PowerPoint slides]. Division of Vital Statistics. https://www.youtube.com/watch?v=2IPJvBMLiqA
